# Comparative genomic, phenotypic, and clinical characterization of ST11-KL25 and ST11-KL64 hypervirulent carbapenem-resistant *Klebsiella pneumoniae* in a tertiary hospital, 2020–2023

**DOI:** 10.1128/aac.01080-25

**Published:** 2026-02-19

**Authors:** Ping Li, Yanghua Xiao, Ruihang Luo, Jingwen Zhang, Feng Nie, TianYu Zou, Yang Liu, Wei Zhang, Tianxin Xiang

**Affiliations:** 1 Jiangxi Provincial Key Laboratory of Respiratory Diseases, Jiangxi Institute of Respiratory Diseases, The Department of Respiratory and Critical Care Medicine, Jiangxi Clinical Research Center for Respiratory Diseases, The First Affiliated Hospital, Jiangxi Medical College, Nanchang University 47861, Nanchang, China; 2 China-Japan Friendship Jiangxi Hospital, National Regional Center for Respiratory Medicine, Nanchang, Jiangxi, China; 3 First Clinical Medical College of Nanchang University, Nanchang University 47861, Nanchang, China; 4 Department of Clinical Microbiology, The First Affiliated Hospital, Jiangxi Medical College, Nanchang University 47861, Nanchang, China; 5 Jiangxi Medical Center for Critical Public Health Events, The First Affiliated Hospital of Nanchang University 117970, Nanchang, Jiangxi, China; Shionogi Inc., Florham Park, New Jersey, USA

**Keywords:** hypervirulent carbapenem-resistant *Klebsiella pneumoniae*, ST11-KL25, antimicrobial resistance, virulence

## Abstract

Hypervirulent carbapenem-resistant *Klebsiella pneumoniae* (hv-CRKP) poses a significant challenge in healthcare settings due to its combination of high virulence and multidrug resistance. In China, ST11 is the predominant lineage, with KL64 and the recently identified KL25 as key capsular subtypes. The clinical and genomic characteristics of ST11-KL25 remain insufficiently characterized. A total of 239 hv-CRKP isolates collected from 2020 to 2023 were analyzed. Molecular typing, antimicrobial susceptibility testing, whole-genome sequencing, and phenotypic assays were used to compare the epidemiology, resistance mechanisms, and virulence traits of ST11-KL25 and ST11-KL64. Clinical data were reviewed to assess infection characteristics. ST11 accounted for 87.9% (210/239) of hv-CRKP isolates. Among ST11 isolates, KL64 (54.7%, 104/190) and KL25 (45.3%, 86/190) were the predominant capsular types. Phylogenetic analysis suggested that ST11-KL25 may have originated from KL64 through capsular switching, with increasing prevalence after 2021. Both sublineages exhibited extensive drug resistance; however, ST11-KL64 showed broader resistance, including higher rates of ceftazidime-avibactam and polymyxin resistance. Phenotypically, ST11-KL25 demonstrated greater competitive fitness and biofilm formation, while ST11-KL64 displayed higher *in vivo* virulence in the *Galleria mellonella* model. ST11-KL25 and ST11-KL64 exhibit distinct adaptation strategies within hv-CRKP. KL25 seems to be a stable, colonization-adapted subtype with consistent resistance and moderate virulence, while KL64 is more invasive, with broader resistance and higher virulence. Subtype-specific surveillance and intervention are essential to limit their spread and protect patient and public health.

## INTRODUCTION

Carbapenem-resistant *Enterobacteriaceae*, particularly carbapenemase-producing *Klebsiella pneumoniae* (CRKP), represent a major global health threat, especially in healthcare-associated infections (1). Since the mid-2000s, the ST258 clone has been a key driver of the global spread of KPC-producing *K. pneumoniae* (2). More recently, the closely related ST11 lineage has rapidly expanded in Asia, now comprising over 60% of CRKP isolates in mainland China and Taiwan (3). The dominance of ST11 CRKP has further restricted treatment options and posed significant challenges for infection control (4).

The recent convergence of multidrug resistance and hypervirulence in *K. pneumoniae* has been associated with increasingly severe clinical outcomes (5). Of particular concern, the ST11 lineage demonstrates a pronounced ability to acquire virulence plasmids, giving rise to hypervirulent carbapenem-resistant *K. pneumoniae* (hv-CRKP) (6). This combination of enhanced virulence and extensive antimicrobial resistance has established ST11-hvCRKP as the predominant epidemic lineage in East Asia, presenting an escalating public health concern (7, 8).

Capsular polysaccharide (CPS), encoded by the highly polymorphic K locus with at least 79 serotypes, is the principal determinant of immune evasion in *K. pneumoniae* (9). Historically, ST11 hv-CRKP isolates in China, typically harboring *blaKPC-2*, were considered to exhibit relatively low virulence (10). This view shifted following reports of fatal outbreaks caused by hypervirulent ST11-KL47 strains carrying pLVPK-like plasmids encoding virulence factors such as *rmpA*2 and *iucABCD* (11, 12). Subsequent genomic analyses revealed that large-scale homologous recombination events at the K and O loci underlie “capsule switching,” reshaping ST11 population structure. Notably, the ST11-KL64 subclone, derived from a KL47-like ancestor, emerged around 2020 with enhanced transmissibility and virulence, rapidly disseminating across China (13, 14). These findings underscore CPS recombination as a key adaptive strategy under host immune and antibiotic pressures, highlighting the urgent need for real-time molecular surveillance (15).

Recently, a novel capsular variant, ST11-KL25, has been sporadically reported in China. Fang et al. described an ST11-KL25 hv-CRKP isolate harboring both bla*NDM*-5 and duplicated *blaKPC-2* alleles alongside multiple virulence plasmids (16). Subsequently, Kang et al. identified another ST11-KL25 strain with a pLVPK-like plasmid exhibiting high lethality in murine models (17). These reports raise concern about the ongoing convergence of extensive drug resistance and hypervirulence in this emerging subclone. However, the clinical prevalence, genomic features, and phenotypic characteristics of ST11-KL25 remain poorly defined.

Routine surveillance at our tertiary hospital in southern China (January 2020 to December 2023) identified ST11-KL25 and ST11-KL64 as the two predominant hv-CRKP sublineages with distinct epidemiological, clinical, and phenotypic profiles. Integrating whole-genome sequencing (WGS) with clinical and phenotypic data, we compared their resistance and virulence landscapes to inform risk stratification, infection control, and region-specific treatment strategies.

## MATERIALS AND METHODS

### Isolate collection and identification

Between January 2020 and December 2023, a total of 1,755 non-duplicate *K. pneumoniae* isolates were obtained from routine clinical specimens processed at the Department of Clinical Laboratory, The First Affiliated Hospital of Nanchang University, Jiangxi, China. The study protocol was reviewed and approved by the Institutional Ethics Committee (Approval No. CDYFY-IACUC-202504GR020). Species identification was conducted using matrix-assisted laser desorption/ionization time-of-flight mass spectrometry (VITEK MS, bioMérieux, Craponne, France), and all isolates were confirmed as *K. pneumoniae*. For long-term preservation, isolates were stored at −80 °C in cryopreservation broth supplemented with 20% (vol/vol) glycerol.

### Antimicrobial susceptibility testing

Carbapenem resistance was initially screened using the Kirby–Bauer disk diffusion method with imipenem and meropenem disks. Isolates exhibiting inhibition zone diameters of ≤19 mm for either drug were interpreted as carbapenem-resistant, in accordance with Clinical and Laboratory Standards Institute guidelines (M100). To confirm resistance, antimicrobial susceptibility testing was subsequently conducted using the agar dilution method in the central laboratory, evaluating susceptibility to ertapenem, imipenem, and meropenem. Isolates showing resistance to at least one of these three agents were classified as CRKP. Based on these criteria, 4,72 isolates were identified as CRKP during the study period (Fig. S1).

### Identification of hypervirulent isolates

Hypervirulent *K. pneumoniae* (HVKP) isolates were identified by PCR detection of at least one virulence gene (*rmpA*, *rmpA*2, *iucA*, *iroB*, and *peg-344*) and a positive string test (viscous string ≥5 mm) (18). Isolates harboring at least one of these genetic markers were defined as HVKP. Isolates with both carbapenem resistance and hypervirulence features were classified as hv-CRKP. Based on these criteria, a total of 239 hv-CRKP isolates were collected between 2020 and 2023 for further investigation. All isolates underwent multilocus sequence typing using seven housekeeping genes (*gapA*, *infB*, *mdh*, *pgi*, *phoE*, *rpoB*, and *tonB*), identifying 15 distinct sequence types (STs). Of these, 190 isolates belonged to the ST11-KL25 or ST11-KL64 lineages and were selected for whole-genome sequencing. The workflow of isolate selection is depicted in Fig. S1.

### Whole-genome sequencing and bioinformatic analysis

Genomic DNA was extracted from overnight cultures of 190 ST11-KL25 and ST11-KL64 hv-CRKP isolates carrying chromosomal mutations using the Bacterial Genomic DNA Extraction Kit (Tiangen Biotech, Beijing, China), following the manufacturer’s protocol. WGS was performed on the Illumina NovaSeq 6000 platform (Illumina, USA), generating paired-end reads with a length of 150 bp. Library preparation was conducted according to standard Illumina procedures. Sequencing reads were assembled *de novo* using SPAdes v4.0.0 with default parameters (19). Capsular (K) and lipopolysaccharide (O) serotypes were identified using Kleborate v3 (20). Phylogenetic relationships among the hv-CRKP isolates were inferred based on single-nucleotide polymorphism (SNP) analysis using Snippy v4.6.0 (https://github.com/tseemann/snippy), with *K. pneumoniae* strain HS11286 (ST11-KL64, GenBank accession no. GCF_000240185.1) serving as the reference genome. A maximum-likelihood phylogenetic tree was constructed using FastTree v2.1.11 (21) under the general time-reversible model and visualized using the Interactive Tree of Life (iTOL, https://itol.embl.de). Antimicrobial resistance genes were detected using ResFinder, plasmid replicon types were identified using PlasmidFinder, and virulence genes were identified using VFDB, all with minimum identity and coverage thresholds of 80% (22–24).

### Phenotypic characterization of ST11-KL25 and ST11-KL64 isolates

To evaluate the phenotypic differences between ST11-KL25 and ST11-KL64, we conducted a series of assays, including growth curve analysis (25), *in vitro* co-culture competition (26), serum-killing assays, *Galleria mellonella* infection experiments (27), and biofilm quantification. For *in vivo* and *in vitro* phenotypic characterization, we selected 20 representative isolates (10 ST11-KL25 and 10 ST11-KL64) from distinct branches of the ST11 phylogenetic tree; one isolate was randomly chosen from each major lineage to ensure broad genetic representation. The specific strains used were ST11-KL64 and ST11-KL25, which are indicated in Table S1. Detailed protocols for all assays are provided in the Supplemental material.

### Statistical analysis

All statistical analyses were performed using SPSS version 26.0 (IBM Corp., Armonk, NY, USA). Categorical variables were compared using the *χ*^2^ test or Fisher’s exact test, as appropriate. Continuous variables were assessed for normality with the Shapiro–Wilk test; normally distributed data were analyzed with Student’s *t*-test or one-way analysis of variance, while non-normally distributed data were evaluated using the Mann–Whitney *U* test or Kruskal-Wallis test. Survival data from *G. mellonella* assays were analyzed using the Kaplan–Meier method and compared by the log-rank (Mantel–Cox) test. Data visualization was performed with GraphPad Prism 8.0 (GraphPad Software Inc., San Diego, CA, USA). Statistical significance was defined as *P <* 0.05, with significance levels indicated as *P < 0.05, **P < 0.01, ***P < 0.001, and ****P < 0.0001.

## RESULTS

### Molecular typing and phylogenetic analysis reveal clonal expansion of ST11-KL25 and ST11-KL64 hv-CRKP lineages

**Fig 1:**
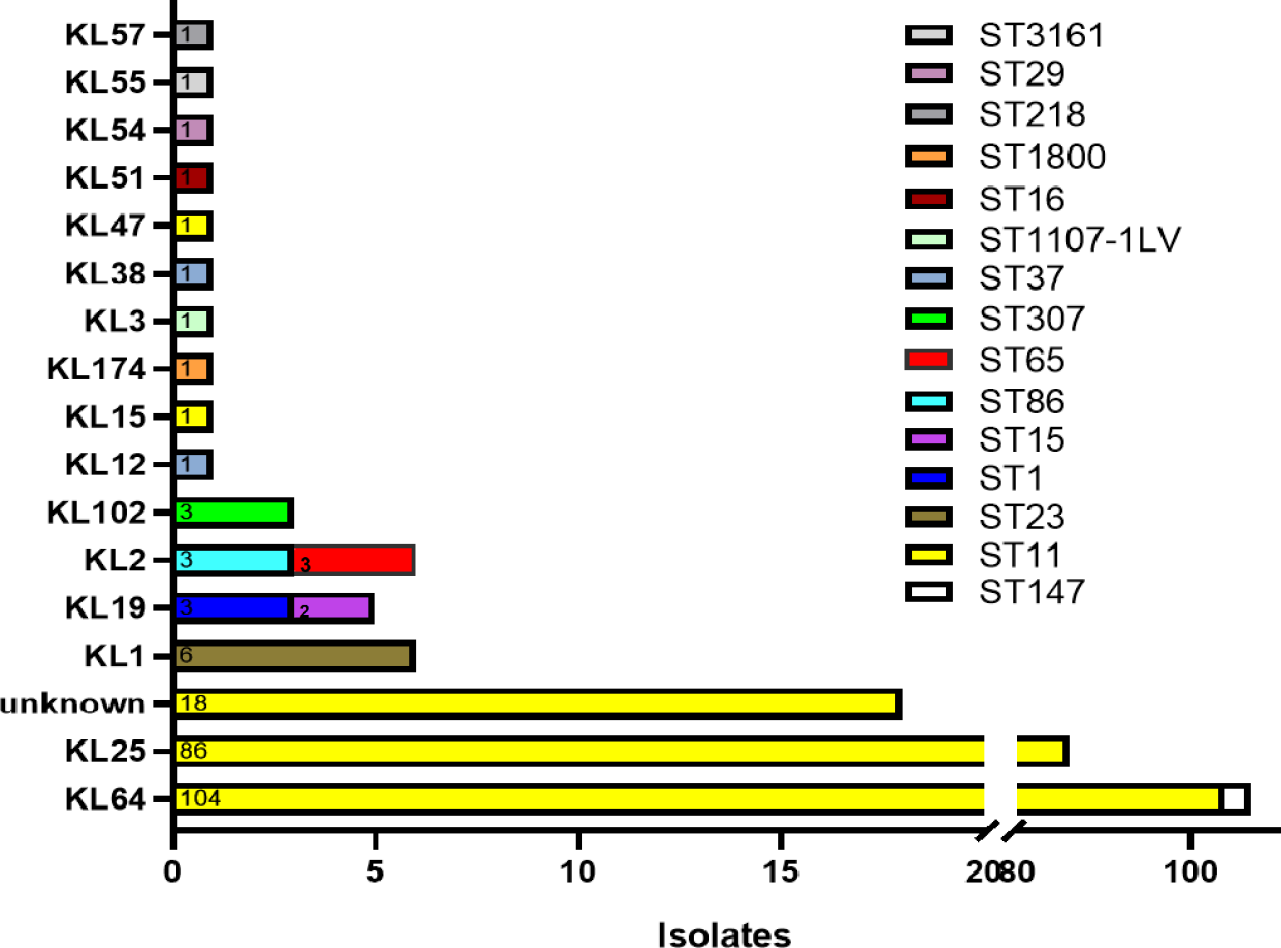
Distribution of STs and capsular locus (K-locus) types among 239 hv-CRKP isolates.

A total of 239 hv-CRKP isolates were subjected to sequence typing, revealing 15 distinct STs. ST11 was the predominant clone, accounting for 87.9% (210/239) of all isolates. Within the ST11 group, the capsular serotypes KL64 (104 isolates) and KL25 (86 isolates) were most frequent, with 18 isolates displaying unclassified K-locus types. Other detected STs included ST23 (2.5%, six isolates), ST1, ST147, ST86, and ST307 (each 1.3%, three isolates), as well as ST15 and ST37 (each 0.8%, two isolates). The remaining sequence types (ST1107-1LV, ST1800, ST218, ST29, ST3161, ST65, and ST16) were each represented by a single isolate (0.4%; Fig. 1; Table S1).

Among the 239 hv-CRKP isolates, 17 different K-locus types were identified. KL64 was the most prevalent, comprising 44.77% (107 isolates), followed by KL25 at 35.98% (86 isolates), and unknown K-loci accounting for 7.53% (18 isolates). Less common capsular types included KL1 (2.51%, six isolates), KL19 (2.09%, five isolates), KL2 (1.67%, four isolates), and KL102 (1.26%, three isolates). The remaining K-loci—KL12, KL15, KL174, KL3, KL38, KL47, KL51, KL54, KL55, and KL57—were each detected in a single isolate (0.42%; Fig. 1; Table S3). Given the dominance of KL25 and KL64 serotypes and the universal presence of the O1/O2v1 O-locus (100%), subsequent analyses focused on comparing the clinical and genomic features of the ST11-KL25 and ST11-KL64 subpopulations. The metadata for 190 ST11-KL64/KL25 hv-CRKP isolates are provided in Table S1.

**Fig 2:**
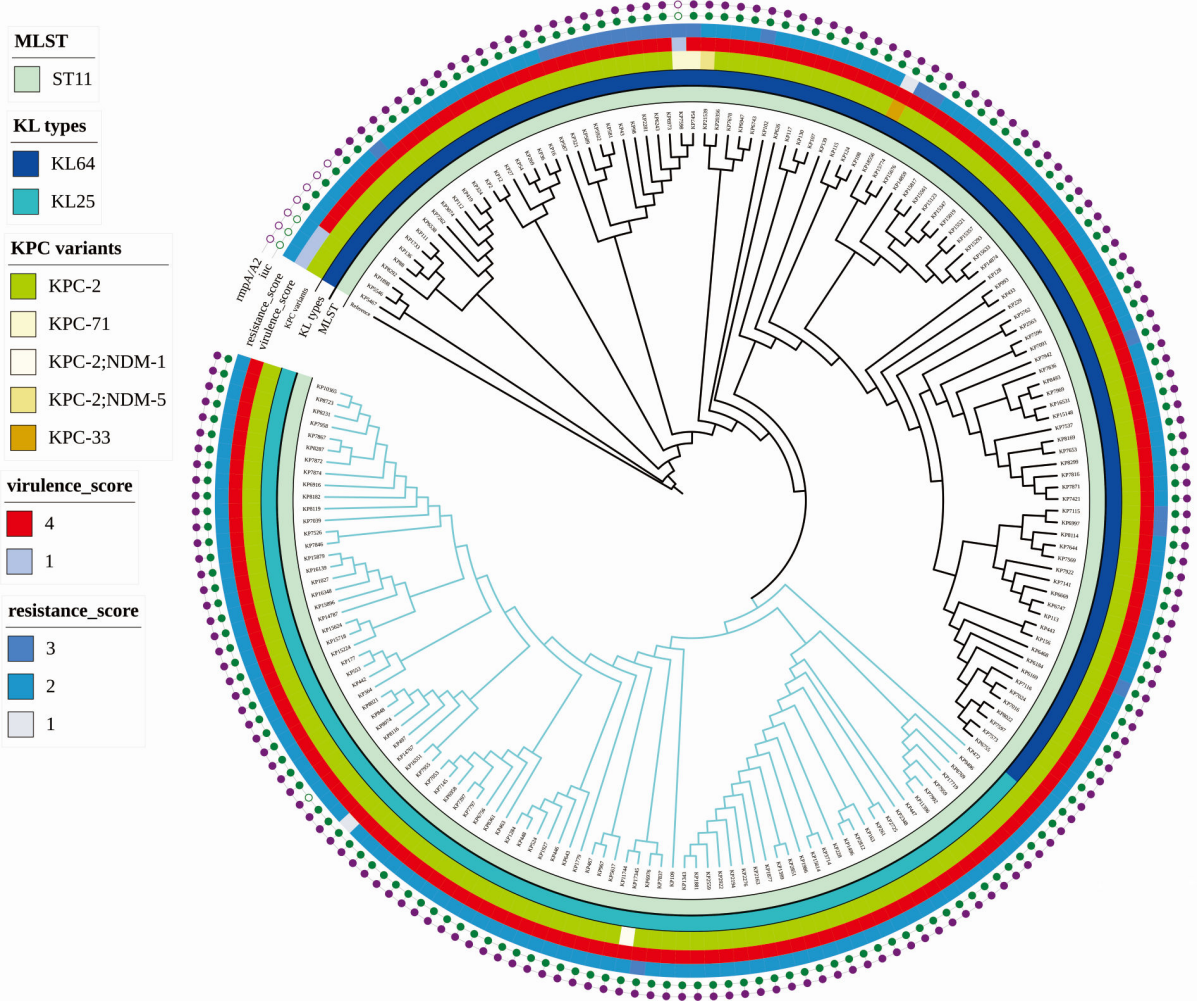
Core genome phylogenetic tree and genomic characteristics of ST11-KL25 and ST11-KL64 hv-CRKP isolates.

A core genome SNP-based phylogenetic analysis of 190 isolates was conducted using strain HS11286 (ST11-KL64) as the reference genome (Fig. 2). The resulting maximum-likelihood phylogenetic tree revealed two major phylogenetic clades that correlated strongly with capsular locus types: KL64 and KL25. The ST11-KL25 isolates (*n* = 86) formed a tightly clustered, monophyletic clade with minimal SNP variation, indicating a clonally expanded lineage with limited genetic diversity. In contrast, ST11-KL64 isolates (*n* = 104) exhibited greater genomic heterogeneity, forming a more dispersed and polyphyletic clade. Notably, the KL25 cluster was embedded within a sublineage of the broader KL64 clade, suggesting that the ST11-KL25 lineage likely originated from a KL64 ancestor through a single capsular switching or recombination event, followed by local clonal expansion.

To further validate the observed differences in genomic diversity between ST11-KL25 and ST11-KL64, we conducted a pairwise core-genome SNP distance analysis for 190 isolates (Fig. S2; Table S2). The analysis revealed a clear distinction between the two lineages: the mean pairwise SNP distance among KL25 isolates was 11.16, compared with 38.20 among KL64 isolates. Statistical analysis using a two-tailed unpaired *t*-test showed that the SNP diversity in KL64 isolates was significantly higher than in KL25 isolates (*t* = 90.76, df = 18,020, *P <* 0.0001). The mean difference was 27.04 ± 0.298, with a 95% CI of 26.46–27.63. An *F*-test further demonstrated significantly different variances between the groups (*F* = 19.72, *P <* 0.0001). These findings demonstrate that the KL25 population represents a tightly clustered, low-diversity lineage, whereas KL64 harbors considerably higher genomic heterogeneity.

### Temporal and clinical distribution of ST11-KL25 and ST11-KL64 hv-CRKP isolates

**Fig 3:**
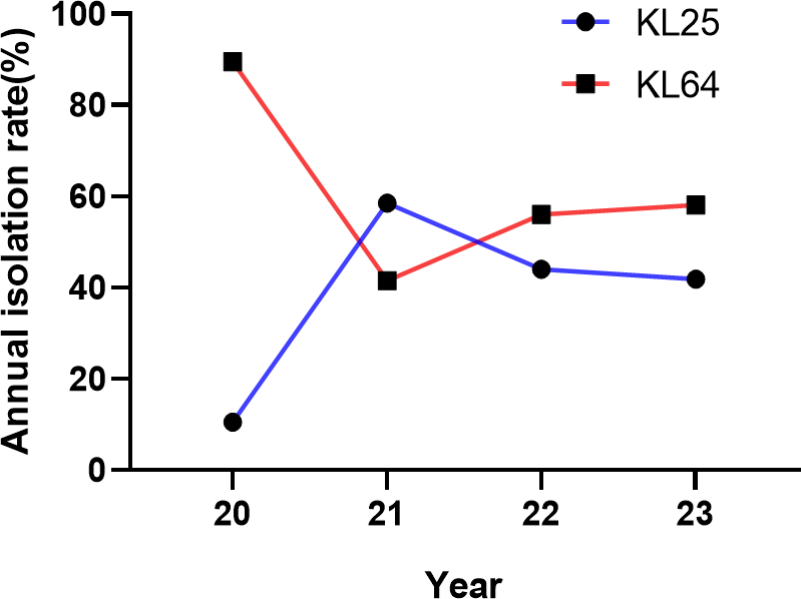
Annual distribution of ST11-KL25 and ST11-KL64 hv-CRKP isolates collected from 2020 to 2023.

A total of 190 ST11-type hv-CRKP isolates belonging to KL25 (*n* = 86) and KL64 (*n* = 104) were collected between 2020 and 2023. The annual distribution within this 190-isolate set was 19 (10.0%) in 2020, 65 (34.2%) in 2021, 75 (39.5%) in 2022, and 31 (16.3%) in 2023. As shown in Fig. 3, ST11-KL64 predominated in 2020 (17/19, 89.5%), whereas a marked increase in ST11-KL25 was observed in 2021 (38/65, 58.5%). In 2022 and 2023, ST11-KL25 and ST11-KL64 coexisted at relatively comparable proportions with minor annual fluctuations.

**Fig 4:**
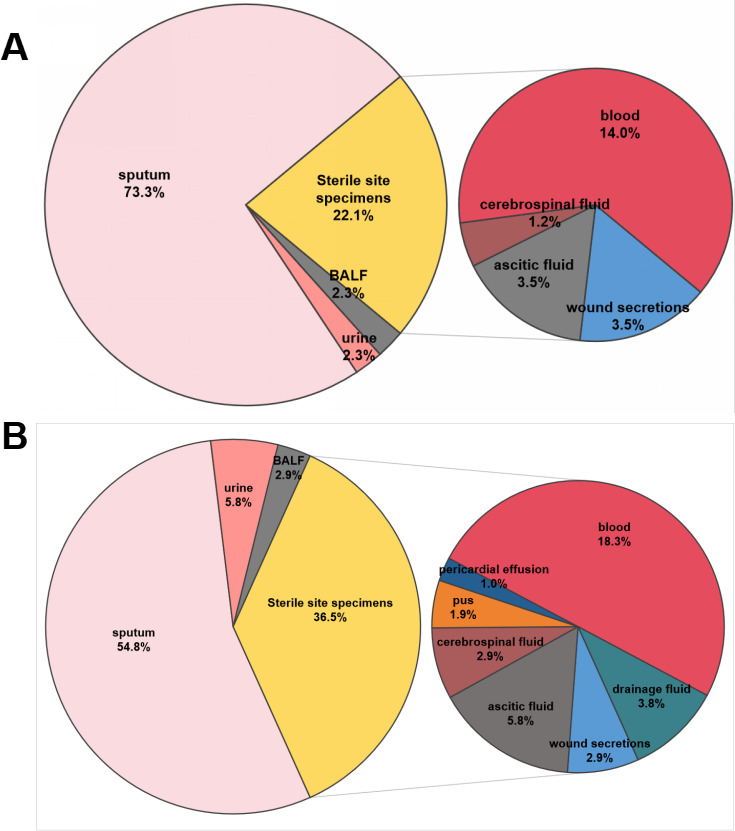
Distribution of clinical specimen sources for ST11-KL25 and ST11-KL64 hv-CRKP isolates. (**A**) ST11-KL25 hv-CRKP isolates. (**B**) ST11-KL64 hv-CRKP isolates.

The distribution of clinical specimen sources differed between ST11-KL25 and ST11-KL64 hv-CRKP isolates (Fig. 4; Table S3). Sputum was the predominant source in both groups and was significantly more frequent among ST11-KL25 than ST11-KL64 isolates (73.3%, 63/86 vs 54.8%, 57/104; *P* < 0.01). The distribution of ST11-KL25 and ST11-KL64 hv-CRKP isolates varied across different clinical departments (Table S4). Among the ST11-KL25 isolates, the majority were obtained from patients in the intensive care unit (ICU; 36.0%, 31/86) and the Department of Neurosurgery (29.1%, 25/86). In contrast, ST11-KL64 isolates were most frequently recovered from the ICU (58.7%, 61/104), followed by the Department of Neurosurgery (15.4%, 16/104). Statistical analysis revealed that the detection rate of ST11-KL64 isolates in the ICU was significantly higher than that of ST11-KL25 (*P <* 0.01), while the detection rate in the Department of Neurosurgery was significantly lower for ST11-KL64 compared to ST11-KL25 (*P <* 0.05). No significant differences were observed between the two subgroups in other departments (*P >* 0.05 for all).

### Comparison of clinical characteristics between patients infected with ST11-KL25 and ST11-KL64 hv-CRKP isolates

Clinical data from patients infected with ST11-KL25 and ST11-KL64 hv-CRKP isolates were analyzed and summarized in Table S5. The majority of patients in both groups were male, with no significant difference in gender distribution (73.3% vs 63.5%, *P >* 0.05). The mean age was similar between the two groups (59.7 vs 59.2 years, *P >* 0.05). Although patients with ST11-KL64 infections had a higher proportion of prolonged hospital stays (>30 days; 59.6% vs 45.3%) and recent hospitalizations within the past month (17.3% vs 13.9%), these differences were not statistically significant (*P >* 0.05). Notably, a significantly higher proportion of patients with ST11-KL64 isolates had a history of ICU admission compared to those with ST11-KL25 (87.5% vs 75.6%, *P <* 0.05). Furthermore, fever was more frequently observed in the ST11-KL64 group (75.0% vs 58.1%, *P <* 0.05).

Other clinical parameters, including infection site (predominantly respiratory tract), time to positive blood culture, antibiotic exposure (both prior to and after strain isolation), prognosis (clinical improvement), and laboratory findings (elevated white blood cell [WBC] count, procalcitonin, and creactiveprotein [CRP] levels), showed no significant differences between the two groups (*P >* 0.05). Regarding underlying comorbidities, the prevalence of hypertension, diabetes, coronary heart disease, stroke, chronic obstructive pulmonary disease (COPD), and malignancy did not differ significantly between the two groups (*P >* 0.05).

### Antimicrobial susceptibility profiles of ST11-KL25 and ST11-KL64 hv-CRKP isolates

**Fig 5:**
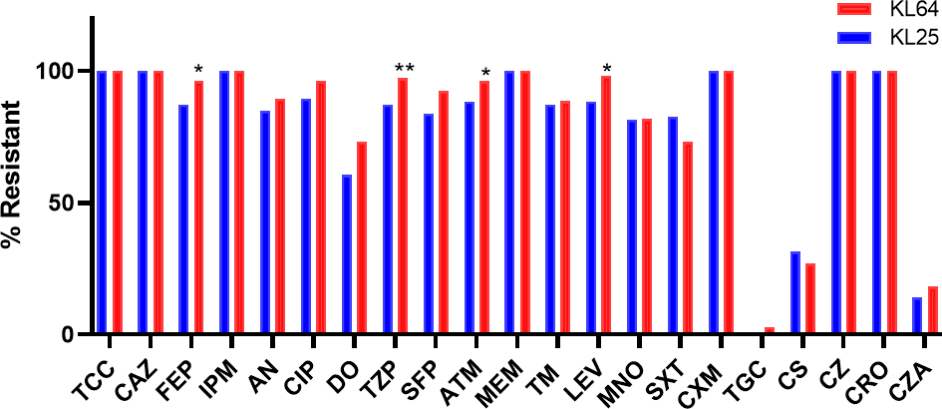
Antimicrobial susceptibility profiles of ST11-KL25 and ST11-KL64 hv-CRKP isolates. Notes: TCC (ticarcillin–clavulanate), CAZ (ceftazidime), FEP (cefepime), IPM (imipenem), AN (amikacin), CIP (ciprofloxacin), DO (doxycycline), CS (colistin), CZ (cefazolin), CRO (ceftriaxone), CZA (ceftazidime–avibactam), TZP (piperacillin–tazobactam), SFP (cefoperazone–sulbactam), ATM (aztreonam), MEM (meropenem), TM (tobramycin), LEV (levofloxacin), MNO (minocycline), SXT (trimethoprim–sulfamethoxazole), CXM (cefuroxime), and TGC (tigecycline). *P < 0.05, **P < 0.01.

Antimicrobial susceptibility profiles are summarized in Fig. 5. Both ST11-KL25 and ST11-KL64 hv-CRKP isolates were extensively drug resistant, with 100% resistance to ceftazidime, imipenem, and meropenem in both groups. The most prominent difference was a markedly higher ceftazidime-avibactam resistance rate in ST11-KL64 than in ST11-KL25 (50.0% vs 13.95%, *P* < 0.001). In addition, ST11-KL64 showed higher resistance to several β-lactams and fluoroquinolones compared with ST11-KL25. Tigecycline remained largely active in both groups.

### Distribution of plasmid replicons in ST11-KL25 and ST11-KL64 hv-CRKP isolates

Plasmid replicon analysis identified 15 distinct types among 86 ST11-KL25 and 104 ST11-KL64 hv-CRKP isolates (Table S6). The two sublineages showed broadly similar plasmid profiles. Five replicons, namely ColRNAI_1, IncFIB(K)_1_Kpn3, IncFII(pHN7A8)1_pHN7A8, IncHI1B_1_p*NDM*-MAR, and IncR_1, were detected at high frequencies (≥98%) in both groups (all *P >* 0.50), indicating a conserved IncF-type plasmid backbone. Several replicons, including Col440I_1, FII (pBK30683)1, IncFIB(pKPHS1)1_pKPHS1, IncFII_1, IncHI2A_1, IncN_1, IncX3_1, and p*ENTA*S02_1, were detected sporadically, with no significant differences between groups (*P >* 0.05). Two replicons showed significant differences between lineages. IncFII(pCRY)_1_pCRY was present in all KL25 isolates but in 93.3% of KL64 isolates (*P* = 0.0168). In contrast, IncFII_1_pKP91 was absent in KL25 and present in 5.8% of KL64 isolates (*P* = 0.0329). In summary, both sublineages share a largely similar IncF-type plasmid structure, with only minor lineage-specific variation.

### Comparative distribution of antimicrobial resistance genes and resistance-associated mutations in ST11-KL25 and ST11-KL64 hv-CRKP isolates

Among 86 ST11-KL25 and 104 ST11-KL64 isolates, both subclones shared a core resistome (*bla*_KPC-2_, quinolone target mutations in GyrA/ParC, and porin alterations in OmpK35/OmpK36; Table S7). *bla*_KPC-2_ was detected in 98.8% of KL25 and 99.0% of KL64 isolates. Notable differences included a higher prevalence of *bla*_CTX-M-65_ in KL25 (100% vs 87.5%) and the presence of *bla*_CTX-M-3_ and *bla*_SHV-182_ only in KL64 (5.8% and 23.1%, respectively), as well as a markedly higher frequency of colistin-associated *mgrB* alterations in KL64 (16.3% vs 1.2%). KL25 also showed higher frequencies of aminoglycoside resistance genes *rmtB* and *aadA2*, whereas *aac(6’)-Ib-cr* and *aadA16* were detected only in KL64. Other resistance determinants that differed between groups are summarized in Table S7. Overall, KL25 showed a more uniform resistance gene profile, whereas KL64 showed greater heterogeneity in resistance genes and mutations.

### Virulence scoring of ST11-KL25 and ST11-KL64 hv-CRKP isolates

After initial screening for classical hvKP markers (*rmpA*, *rmpA*2, *iucA*, *iroB*, and *peg-344*), whole-genome sequencing data were analyzed using Kleborate, which assigns virulence scores based on the presence of *yersiniabactin (ybt)*, *colibactin (clb)*, and *aerobactin (iuc)*. Kleborate virulence scoring showed a comparable baseline virulence genotype in the two subclones: all ST11-KL25 isolates (100%, 86/86) carried both ybt and iuc (score 4), and most ST11-KL64 isolates (96.2%, 100/104) had the same score, while a small subset of KL64 carried ybt alone (3.8%, 4/104; score 1).

A comprehensive genomic analysis of virulence-associated genes was conducted for ST11-KL25 and ST11-KL64 hv-CRKP isolates (Table S8). Both groups showed 100% prevalence of key siderophore and iron acquisition genes, including *fyuA*, *irp1/2*, *iucABCD*, *iutA*, and *ybtA-X*, as well as universal presence of enterobactin genes (*entA* and *entB*), *fepC*, *ompA*, and all ecp-encoded fimbriae. Differences were observed in the structure of the yersiniabactin-associated *ICEKp3* genomic island. The complete *ybt9-ICEKp3* structure was more frequent in KL25 (97.7%) than in KL64 (82.7%; *P <* 0.001), while truncated *ICEKp3* forms were more common in KL64 (15.4% vs 1.2%, *P <* 0.001). *YbST* analysis indicated that KL25 was dominated by *YbST* 503-1LV (96.5%), whereas KL64 showed greater subtype diversity, including *YbST* 503, 503-2LV, and 503-3LV (*P <* 0.001). No isolates harbored *colibactin*, *salmochelin*, or the corresponding biosynthetic clusters. The heat-stable enterotoxin gene *astA* was detected only in KL64 isolates (5.8%, *P* = 0.0329).

Variation was also noted in *rmp*-associated loci. The canonical *rmpADC* operon and *rmpA*2 were absent in KL25 but variably present in KL64. Both truncated and complete *rmp1-KpVP*-1 alleles were found only in KL64 (*P <* 0.001). KL25 isolates were exclusively *RmST* type 0, while KL64 exhibited multiple *RmST* types, such as *RmST* 0,40 and 0,26-1LV (each 9.6%, *P* = 0.002). The partial allele *rmpA*2_3-47% was slightly more common in KL25 (98.8% vs 92.3%, *P* = 0.048), whereas the functional *rmpA*2 gene was only detected in 7.7% of KL64 isolates (*P* = 0.009). In summary, ST11-KL25 isolates had a conserved virulence gene profile, characterized by an intact *ybt9-ICEKp3* element. In contrast, ST11-KL64 isolates demonstrated greater genetic heterogeneity, with more diverse rmp variants and the presence of *astA* in a subset of isolates.

### ST11-KL25 exhibits enhanced competitive fitness and biofilm formation, while ST11-KL64 demonstrates higher virulence

**Fig 6:**
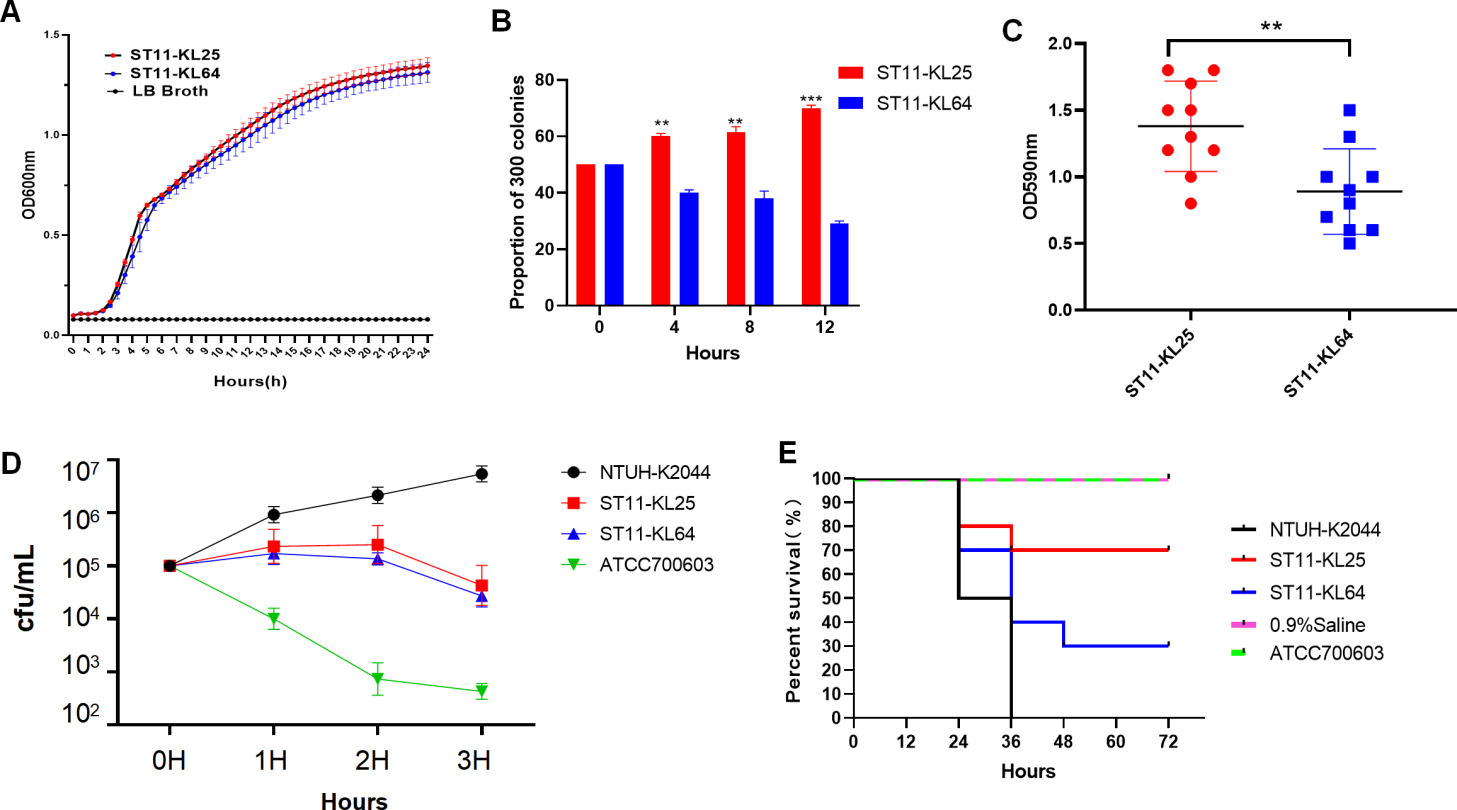
Virulence-associated phenotypes of ST11-KL25 and ST11-KL64 isolates. (**A**) Growth curve analysis illustrated the growth dynamics of ST11-KL25, ST11-KL64, and LB broth at 37°C. (**B**) *In vitro* competition assays demonstrated the competitive fitness of ST11-KL25 and ST11-KL64 at 0, 4, 8, and 12 h under antibiotic-free conditions. (**C**) Biofilm assays indicated a significantly higher proportion of ST11-KL25 isolates with moderate/strong biofilm forming capacity compared to ST11-KL64. (**D**) Serum killing assays evaluated the survival ability of NTUH-K2044, ST11-KL25, ST11-KL64, and ATCC 700603 in pooled human serum. (**E**) The *G. mellonella* infection model was employed to assess the *in vivo* virulence of NTUH-K2044, ST11-KL25, ST11-KL64, and ATCC700603. **P < 0.01, ***P < 0.001.

A series of phenotypic assays were conducted to compare ST11-KL25 and ST11-KL64 isolates, including growth kinetics, competitive fitness, biofilm formation, serum resistance, and *G. mellonella* infection-based virulence testing. Growth curve analysis showed that both groups had similar lag and exponential phases in LB broth at 37°C, entering the stationary phase at 6 h (Fig. 6A). However, during the stationary phase (8–24 h), KL25 maintained significantly higher OD600 values than KL64 (*P <* 0.05 at all timepoints). Competition assays demonstrated that ST11-KL25 had a higher relative growth rate and outcompeted ST11-KL64 over time (*P <* 0.01, Fig. 6B). At each timepoint after 4 h, ST11-KL25 progressively dominated the colony proportion. Biofilm assays revealed that a significantly higher proportion of ST11-KL25 isolates produced strong biofilms compared to ST11-KL64 (*P <* 0.01; Fig. 6C). Crystal violet staining and OD590 nm measurements indicated greater biofilm biomass in ST11-KL25. Serum killing assays indicated that both sublineages had moderate serum resistance, with survival rates higher than the classical reference strain *K. pneumoniae* ATCC700603 but lower than the hypervirulent strain NTUH-K2044 (Fig. 6D). In the *G. mellonella* infection model, both groups caused significantly higher larval mortality than the PBS control (*P <* 0.001; Fig. 6E). ST11-KL64 isolates resulted in over 70% mortality, whereas ST11-KL25 caused less than 30% mortality. Both groups were less virulent than NTUH-K2044, which caused 100% mortality within 36 h. In summary, ST11-KL25 isolates demonstrated enhanced competitive fitness and biofilm formation, while ST11-KL64 showed higher *in vivo* virulence in the *G. mellonella* model.

## DISCUSSION

CRKP, particularly hv-CRKP, poses a significant public health threat, especially in healthcare settings (28). In China, the ST11 lineage is the predominant clone, exhibiting considerable diversity in capsular types, resistance mechanisms, and virulence factors (4, 7). The emergence of the novel ST11-KL25 subclone, alongside the established ST11-KL64, underscores the ongoing adaptive evolution within hv-CRKP. While ST11-KL64 is well recognized as a high-risk clone (29), the molecular and phenotypic characteristics of ST11-KL25 have remained largely uncharacterized. This study provides a comprehensive analysis of ST11-KL25, detailing its epidemiology, genomic structure, and phenotypic features in comparison to ST11-KL64.

ST11-KL25 exhibited high clonality, limited SNP diversity, and a uniform O1/O2v1 antigen profile, forming a distinct monophyletic clade in core-genome SNP analysis. Phylogenetic proximity to a defined sublineage within the genetically diverse ST11-KL64 population suggests that ST11-KL25 likely originated from a KL64 ancestor via a single capsule-switching or homologous recombination event, followed by clonal expansion. This is further supported by the increasing prevalence of ST11-KL25 since 2021, indicating successful dissemination of this genetically conserved subclone. In contrast, ST11-KL64 demonstrates substantial genomic plasticity, with greater genetic variability, diverse resistance determinants, porin gene mutations, and *ICEKp3* rearrangements—hallmarks of ongoing adaptation under antimicrobial pressure. These findings extend previous reports of virulent KL25 strains and provide important insights into the epidemiological and genetic landscape of this emerging subclone (16, 17). Collectively, these results highlight ST11-KL25 as a recently emerged and clonally expanding lineage with a restricted repertoire of resistance and virulence genes, whereas ST11-KL64 remains a dynamic, evolving population with broad adaptive potential.

Distinct resistance patterns were observed between the two subclones. ST11-KL25 displayed a relatively conserved resistome, characterized by the presence of *bla*_KPC-2_, *bla*_CTX-M-65_, *bla*_SHV-12_, and consistent porin alterations (*OmpK*36-GD mutation and *OmpK*35 deletion). In contrast, ST11-KL64 exhibited a more complex and variable resistance profile, frequently carrying additional ESBL genes (*bla*_CTX-M-3_ and *bla*_CTX-M-14_), fluoroquinolone and rifampin resistance markers (*aac(6′)-Ib-cr*, *arr-3*, *qnr*, and *sul1/2*), and *mgrB* disruptions associated with colistin resistance (30).

Virulence profiling further differentiated the two subclones. A subset of ST11-KL64 isolates retained complete iuc1 *aerobactin* operons and *rmpADC*/*rmpA*2 regulators, associated with high mucoviscosity, serum resistance, and >70% lethality in *G. mellonella* infection models. In contrast, all ST11-KL25 isolates lacked the *rmp* loci but retained *ybt9-ICEKp3* in 97.7% of strains. Although ST11-KL25 displayed lower lethality, it was associated with strong biofilm formation, indicating a low-virulence, high-adherence phenotype that may facilitate persistent colonization. This enhanced biofilm-forming ability may be related to differences in capsular composition and CPS biosynthesis loci between KL25 and KL64, though the molecular mechanisms warrant further investigation. Fitness assays further demonstrated the superior growth competitiveness of ST11-KL25. Together with its enhanced biofilm formation and the lower lethality observed in the *G. mellonella* model compared with ST11-KL64, these findings suggest that ST11-KL25 may be better adapted for colonization and long-term persistence in the hospital niches rather than for causing acute invasive disease. A similar colonization-favored adaptation has been reported for the ST11-KL47 lineage (12, 31).

Clinically, ST11-KL25 and ST11-KL64 showed different clinical sampling-source distributions and patient-setting associations. ST11-KL25 was predominantly recovered from respiratory specimens, particularly sputum (73.3%), and was more frequently identified in neurosurgical patients, which may reflect an increased propensity for airway colonization and/or reflect sampling practices. In contrast, ST11-KL64 was more often recovered from sterile sites and was closely associated with ICU admission and septic presentations. These findings highlight the clinical significance of ST11-KL64 as an invasive and virulent subclone (32). ST11-KL25 was detected across multiple departments over 4 years (2020–2023), without evidence of temporal or spatial clustering, and had a lower ICU proportion compared to ST11-KL64. These observations suggest that the differing distributions of the two subclones are primarily attributable to their intrinsic biological characteristics and ecological adaptation, rather than localized outbreak events.

Our epidemiological and phenotypic data suggest different clinical associations for the two subclones. ST11-KL64 was enriched in ICU settings and more frequently recovered from sterile sites, consistent with adaptation to healthcare environments characterized by intense antimicrobial exposure and invasive procedures, and with a more invasive clinical phenotype. In contrast, ST11-KL25 was more frequently recovered from respiratory (non-sterile) specimens and showed stronger biofilm formation/competitive fitness, which may facilitate colonization and persistence in the host and hospital environment. Similar colonization–invasion trade-offs and dynamic gain/loss of virulence determinants have been proposed for ST258 sublineages (33, 34).

This study has several limitations. First, it was conducted at a single center; although temporal and spatial analyses did not suggest ward-level outbreaks, localized clonal expansion cannot be excluded. Second, the low genomic diversity of ST11-KL25 may reflect its recent emergence, and the limited number of ST11 isolates reduces phylogenetic resolution. Multi-center genomic data sets will be needed to validate these evolutionary patterns.

In summary, ST11-KL25 and ST11-KL64 appear to follow distinct strategies within hv-CRKP. ST11-KL64 carries multiple virulence and resistance determinants and is more often linked to invasive infections and ICU settings. In contrast, ST11-KL25 shows stronger biofilm formation and is more frequently recovered from non-sterile sites, consistent with increased colonization potential. Despite lower virulence and resistance, persistent colonization by KL25 may facilitate ongoing hospital circulation. These findings underscore heterogeneity within ST11 hv-CRKP and support subclone-level surveillance with real-time genomic monitoring and targeted screening of key resistance and virulence markers.

### Conclusion

This study presents a comparative analysis of ST11-KL25 and ST11-KL64 hv-CRKP subclones from a single center in China. ST11-KL25 exhibited lower virulence but stronger colonization capacity, while ST11-KL64 showed higher invasiveness and greater genomic diversity. The coexistence of these subclones underscores the heterogeneity within the ST11 lineage and highlights the importance of subclone-level genomic surveillance. Ongoing monitoring will be essential for tracking transmission and informing infection control strategies.

## Data Availability

The Illumina sequences of all isolates are available in the NCBI database (accession no. PRJNA1261662).

## SUPPLEMENTAL MATERIAL

Supplemental material aac.01080-25-s0001.docx Methods; Fig. S1 and S2.

Table S1 aac.01080-25-s0002.xlsx Metadata of 190 ST11-KL64/KL25 hv-CRKP isolates.

Table S2 aac.01080-25-s0003.xlsx Pairwise SNP distance matrix of 190 hv-CRKP Isolates.

Table S3 aac.01080-25-s0004.xlsx Distribution of clinical specimen sources for ST11-KL25 and ST11-KL64 hv-CRKP isolates.

Table S4 aac.01080-25-s0005.xlsx Distribution of ST11-KL25 and ST11-KL64 hv-CRKP isolates among clinical departments.

Table S5 aac.01080-25-s0006.xlsx Comparison of clinical characteristics between patients infected with ST11-KL25 and ST11-KL64 hv-CRKP isolates.

Table S6 aac.01080-25-s0007.xlsx Distribution of plasmid replicons in ST11-KL25 and ST11-KL64 hv-CRKP isolates.

Table S7 aac.01080-25-s0008.xlsx Comparative distribution of antimicrobial resistance genes and resistance-associated mutations in ST11-KL25 and ST11-KL64 hv-CRKP isolates.

Table S8 aac.01080-25-s0009.xlsx Distribution of virulence genes and associated sequence types in ST11-KL25 and ST11-KL64 hv-CRKP isolates.

ASM does not own the copyrights to Supplemental Material that may be linked to, or accessed through, an article. The authors have granted ASM a non-exclusive, world-wide license to publish the Supplemental Material files. Please contact the corresponding author directly for reuse.
