# Supplementary material for: Comparative genomic, phenotypic, and clinical characterization of ST11-KL25 and ST11-KL64 hypervirulent carbapenem-resistant Klebsiella pneumoniae in a tertiary hospital, 2020–2023
Source: Antimicrob Agents Chemother. 2026 Feb 19;70(4):e01080-25. doi: 10.1128/aac.01080-25 (PMC13041405; doi:10.1128/aac.01080-25)

**Supplementary Materials**

**Materials and Methods**

**Growth Curve Assay and *in vivo* competition experiment**

Bacterial growth dynamics were assessed using the 20 representative isolates selected as described above. For the growth curve assay, the cultures were incubated at 37 °C, and optical density at 600 nm (OD600) was measured every 5 minutes using a microplate reader. Growth rates were calculated from OD600 values using custom R scripts. To assess the relative competitive fitness of ST11-KL25 and ST11-KL64 under identical *in vivo* conditions, we performed a co-culture competition assay, aiming to determine whether ST11-KL25 possesses a fitness advantage that may facilitate its further expansion or replacement of ST11-KL64 in clinical settings. In the *in vivo* competition assay, bacterial suspensions were adjusted to a concentration of 0.5 × 10⁶ CFU/mL. Equal volumes of isolates with distinct capsular serotypes were mixed and co-cultured in LB broth under shaking conditions (200 rpm, 37 °C). At defined time points (0, 4, 8, and 12 hours), samples were collected, serially diluted in phosphate-buffered saline (PBS), and plated on LB agar. ST11-KL64 carries the *wzi64* allele, whereas ST11-KL25 lacks a corresponding *wzi* allele due to its KL25 capsular locus structure; therefore, *wzi64* was used as a KL64‑specific molecular marker to distinguish the two lineages in mixed cultures. A 580-bp fragment of the *wzi64* gene was amplified by PCR using established protocols^22^. For each time point and each biological replicate, 300 colonies were randomly selected, and each colony was subjected to PCR to determine whether it belonged to ST11‑KL25 (*wzi64*‑negative) or ST11‑KL64 (*wzi64*‑positive). The experiment was performed in three independent biological replicates, and the proportions of KL25 and KL64 were calculated from the PCR results to quantify changes in capsular serotype prevalence over time.

**Serum-Killing Assay**

To further evaluate bacterial virulence, a serum bactericidal assay was conducted using the 20 representative isolates selected as described above. Log-phase bacterial cultures were adjusted to 1 × 10⁶ CFU/mL, and 25 μL of the suspension was mixed with 75 μL of pooled human serum in a 96-well microtiter plate. The mixtures were incubated at 37 °C, and aliquots were taken at 0, 1, 2, and 3 hours for quantification of viable bacteria via serial dilution and plating. All assays were performed in triplicate, and bacterial survival was expressed as CFU/mL at each time point.

***Galleria mellonella* Infection Model**

The *in vivo* virulence of bacterial strains was assessed using the *G.mellonella* infection model with the 20 representative isolates selected as described above^23^. Log-phase bacterial cultures were prepared at concentrations of 5 × 10⁸, 5 × 10⁶, and 5 × 10⁴ CFU/mL, washed twice with PBS, and diluted to working suspensions. Groups of 10 larvae were each injected with 10 µL of the respective bacterial suspension and incubated at 37 °C in the dark. Larval survival was recorded daily for up to 72 hours post-infection. All experiments were performed in triplicate.

**Biofilm Formation**

The biofilm-forming ability of isolates was evaluated using a microtiter plate assay with the 20 representative isolates selected as described above. Overnight bacterial cultures were diluted 1:100 in 200 μL of Mueller-Hinton (MH) broth and incubated in polystyrene 96-well plates at 37 °C for 24 hours. Following incubation, biofilms were quantified by crystal violet staining and measurement of OD at 590 nm. Each experiment was performed in triplicate. All experiments were performed in triplicate.

Figure S1. Flowchart of isolate selection in this study.


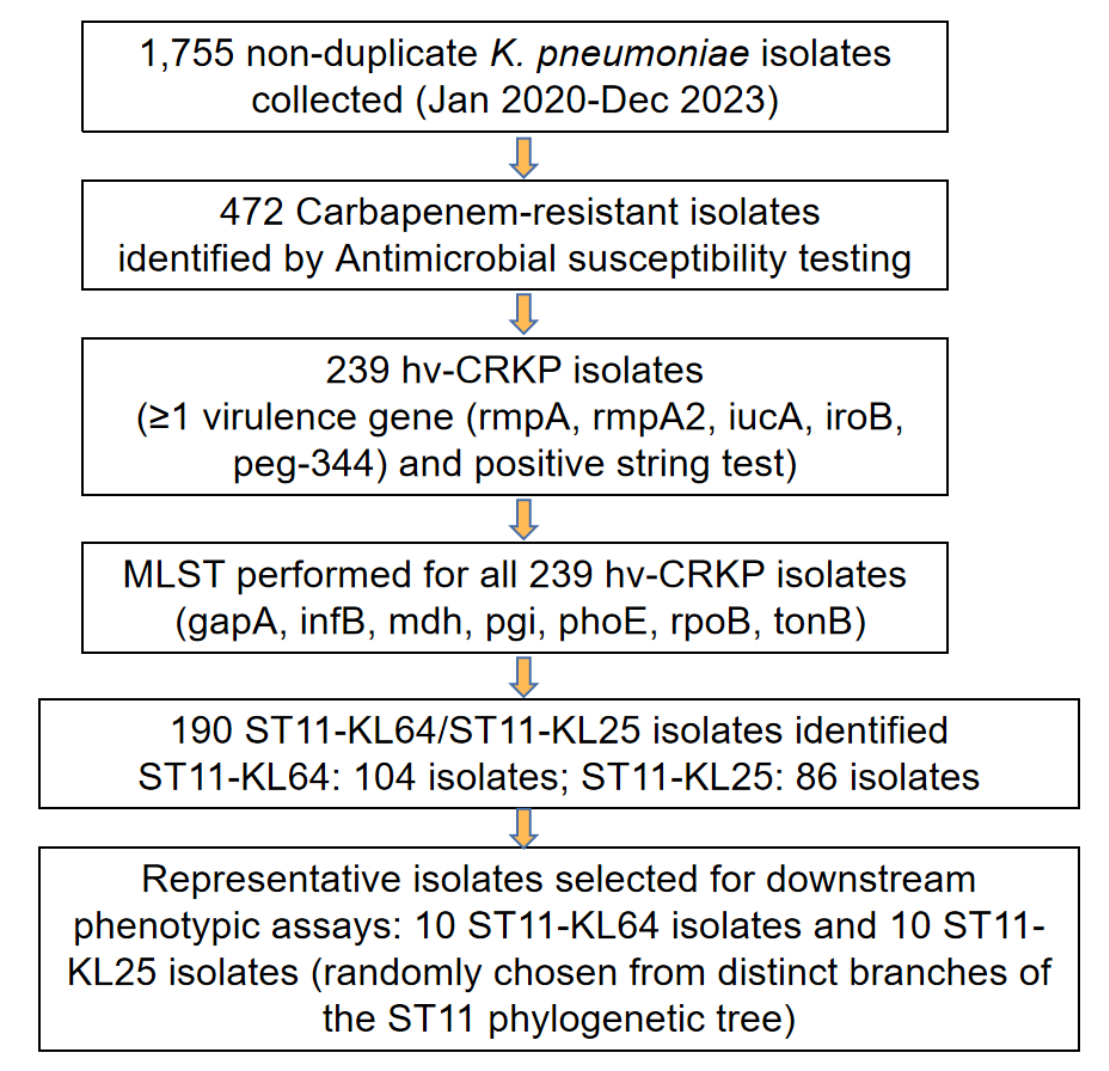


Figure S2. Pairwise SNP distance heatmap of ST11-KL25 and ST11-KL64 isolates.


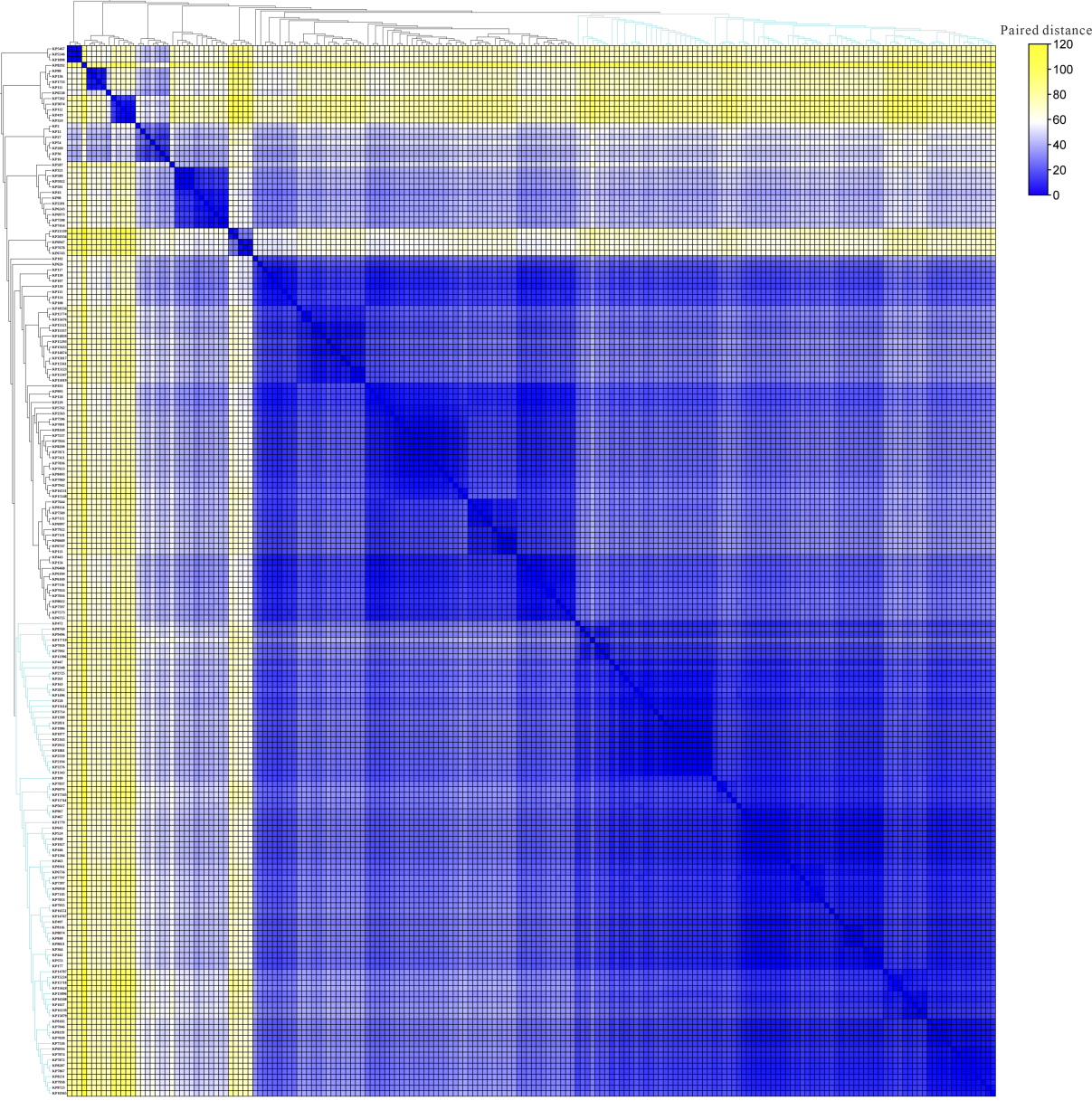

Supplement: Supplemental material — Methods; Fig. S1 and S2. [file aac.01080-25-s0001.docx]
